# Differential Phenotypic and Functional Profiles of TcCA-2 -Specific Cytotoxic CD8^+^ T Cells in the Asymptomatic versus Cardiac Phase in Chagasic Patients

**DOI:** 10.1371/journal.pone.0122115

**Published:** 2015-03-27

**Authors:** Adriana Egui, M. Carmen Thomas, Bartolomé Carrilero, Manuel Segovia, Carlos Alonso, Concepción Marañón, Manuel Carlos López

**Affiliations:** 1 Instituto de Parasitología y Biomedicina López Neyra, Consejo Superior de Investigaciones Científicas (IPBLN-CSIC), PTS Granada, Avda. del Conocimiento S/N, 18016, Granada, Spain; 2 Unidad Regional de Medicina Tropical, Hospital Virgen de la Arrixaca, Carretera Madrid-Cartagena s/n, El Palmar, 30120, Murcia, Spain; 3 Centro de Biología Molecular Severo Ochoa, CSIC-Universidad Autónoma de Madrid, Cantoblanco, Madrid, Spain; 4 Genomic Medicine Department, Centre for Genomics and Oncological Research (GENYO): Pfizer / University of Granada / Andalusian Regional Government, PTS Granada, Spain; Centro de Investigacion y de Estudios Avanzados del Instituto Politecnico Nacional, MEXICO

## Abstract

It has been reported that the immune response mediated by T CD8^+^ lymphocytes plays a critical role in the control of *Trypanosoma cruzi* infection and that the clinical symptoms of Chagas disease appear to be related to the competence of the CD8^+^ T immune response against the parasite. Herewith, *in silico* prediction and binding assays on TAP-deficient T2 cells were used to identify potential HLA-A*02:01 ligands in the *T*. *cruzi* TcCA-2 protein. The TcCA-2-specific CD8^+^ T cells were functionality evaluated by Granzyme B and cytokine production in peripheral blood mononuclear cells (PBMC) from Chagas disease patients stimulated with the identified HLA-A*02:01 peptides. The specific cells were phenotypically characterized by flow cytometry using several surface markers and HLA-A*02:01 APC-labeled dextramer loaded with the peptides. In the *T*. *cruzi* TcCA-2 protein four T CD8^+^ epitopes were identified which are processed and presented during Chagas disease. Interestingly, a differential cellular phenotypic profile could be correlated with the severity of the disease. The TcCA-2-specific T CD8^+^ cells from patients with cardiac symptoms are mainly effector memory cells (T_EM_ and T_EMRA_) while, those present in the asymptomatic phase are predominantly naive cells (T_NAIVE_). Moreover, in patients with cardiac symptoms the percentage of cells with senescence features is significantly higher than in patients at the asymptomatic phase of the disease. We consider that the identification of these new class I-restricted epitopes are helpful for designing biomarkers of sickness pathology as well as the development of immunotherapies against *T*. *cruzi* infection.

## Introduction

*Trypanosoma cruzi* is the etiological agent of the Chagas disease (ChD), which affects at least 8 million people in Central and South America [1]. In this geographic area more than 25 million people are at risk of infection. The increasing number of migrants from Latin-American countries has globally spread the *T*. *cruzi* infection to non-endemic areas. Thus, the ChD represents an important global health problem [2,3]. The disease courses with various clinical forms. The acute phase appears shortly after infection. In the absence of treatment, the acute phase is followed by an asymptomatic phase in which the parasites are present into specific tissues [4]. In about 30% of patients, the infection leads to a symptomatic chronic phase, characterized by severe cardiac and digestive involvements [5,6].

To date, the efficacy of the current and the rather toxic anti-parasitic chemotherapy is under concern in patients in the chronic phase of the disease [7,8]. Based on clinical and immunological evidence, the World Health Organization and several scientific networks recommend the use of anti-parasite treatments in all chronic-phases of *T*. *cruzi* infected individuals [9]. However, the efficacy of the available current drugs against the chronic phase of the disease is under study. Likewise, recent clinical trials with new drugs such as azoles are disappointing [10,11]. In addition, vaccines or immunotherapeutic agents for prevention and treatment of *T*. *cruzi* infection are practically non-existing [12]. Given the magnitude of the disease, accurate and safe therapeutic agents able to control the infection are extremely urgent.

The effector functions mediated by T lymphocytes are essential for the control of the parasite proliferation. Thus, the initial activation of CD4^+^ T lymphocytes, the subsequent activation and proliferation of T CD8^+^ lymphocytes and the activation of B lymphocytes play a crucial role in the control of the parasite replication [13]. In experimental models of infection it has been shown that the induction of cellular immunity and, particularly, the response mediated by T CD8^+^ lymphocytes is crucial for the control of *T*. *cruzi* proliferation [14,15]. Although several parasite class I-restricted antigenic epitopes have been characterized in murine experimental models [16], the CD8^+^ response in Chagas patients is limited to a few epitopes [17–21]. In fact, the information about the function and phenotype of CD8^+^ T cells recognizing these few epitopes is very incomplete [22]. It is also known that during *T*. *cruzi* infection the parasite restricts the repertoire of CD8^+^ T cells that generate strong immunodominance [23]. It has been suggested, in addition, that the immunological restriction is a mechanism probably used by the parasite to reduce the magnitude of the immune response of the host favoring, thus, parasitism [23]. It seems, furthermore, that during natural infection the immune response is not strong enough to attain sterility since the parasites persist hidden into particular patient´s tissues [24,25].

The parasite clearance observed in treated mice results in the emergence of a stable, parasite-specific CD8^+^ T cell population with the characteristic of central memory cells, based upon expression of CD62L, CD122 and CD127 [26]. Many criteria have been used to characterize the antigen-experienced memory CD8^+^ T cells including the expression of the activation marker CD45RA, the homing receptor CCR7, the co-stimulatory molecule CD27 and the IL7 cytokine receptor (CD127) [27–29]. The CD127^+^ antigen-specific CD8^+^ T cells present in *T*. *cruzi* infected mice produce IFN-γ after peptide re-stimulation and are persistent when transferred to an antigen-free environment [30]. Since a significant higher clinical score has been observed in patients with low CD8^+^ T lymphocytes able to produce IFN-γ in response to *T*. *cruzi* parasite it seems that the clinical symptoms of Chagas disease are directly related to the efficiency of the immune response against the parasite. In individuals having a severe chronic *T*. *cruzi* infection the low CD8^+^ T cells count has been mainly associated to an increased differentiation of memory CD8^+^ T cells (CD27^-^CD28^-^CD45RA^-^) as well as to a clonal deployment [31].

In the present paper we describe the identification of four T CD8^+^ epitopes restricted to the HLA-A*02:01 molecule in the *T*. *cruzi* TcCA-2 protein. The TcCA-2 protein, as well as the homologous TCR39 and B13 antigens, contains a track of N-terminal 12 aa imperfect repetition motif [32,33], which seems to possess a significant degree of antigenicity and to be a target of B-cell responses. Actually, the repetition motifs are being used as diagnostic tools [32,34,35], as well as to assess the severity of the disease [36]. It has also been described that the repetitive domains existing in the B13 antigen is recognized by T cells in the context of several HLA class II molecules [37]. The four newly identified T CD8^+^ epitopes are successfully processed and presented during ChD. The phenotype and functional activity of the identified peptide-specific CD8^+^ T cells from chagasic patients, at different phases of the disease, are also analyzed. Remarkably, a differential phenotypic profile has been observed relative to the severity of the disease. Thus, the TcCA-2-specific CD8^+^ T cells from patients with cardiac symptoms are mainly effector memory cells (T_EM_ and T_EMRA_) while, those present in patients at the asymptomatic phase are predominantly naive cells (T_NAIVE_). Moreover, in patients with cardiac symptoms the percentage of cells with senescence features (CD44^+^/CD57^+^) is higher than that detected in patients at the asymptomatic phase of the disease.

## Materials and Methods

### Prediction of HLA-A*02:01 epitopes and synthetic peptides

The prediction of potential HLA-A*02:01 ligands in the *T*. *cruzi* TcCA-2 protein was performed by analyzing the deduced amino acid sequence of the TcCA-2 gene (GenBank, CAI strain, accession number M92049.1 and CL Brenner strain, accession number XM813834) following the criteria described by Rammense et al [38]. For the selected epitopes, two *in silico* HLA-A*02:01 binding prediction algorithms where used, SYFPEITHI to analyze the affinity (www.syfpeithi.de) and BIMAS to calculate the stability (half-life) of the complex (www-bimas.cit.nih.gov). These epitopes presented the lowest scores using the Immune Epitope Database and Analysis Resource (IEDB), where a low score is associated to a high binding affinity (http://tools.immuneepitope.org/main/html/references.html).

Peptides bearing the described HLA-A*02:01 binding motifs were synthesized by simultaneous multiple-peptide solid-phase methods. The peptides were assembled using the standard t-Boc solid-phase-peptide synthesis (SPPS) strategy on a P-metylbenzhydrilamide (MBHA) resin. Purity was checked by high performance liquid chromatography (HPLC), and the correct composition of the peptide was verified by mass spectrometry [39]. Peptides were dissolved to 1 mM final concentration in water containing 10% DMSO and stored at -20°C.

### Cell lines

TAP-deficient T2 cells [40] were used for the HLA-A*02:01 binding assays and the K562-A2 cells [41] for the HLA-A*02:01 restriction assay. The cells were cultured in complete RPMI medium supplemented with 10% of inactivated Fetal Calf Serum (iFCS), 2 mM glutamine, and 100 U/ml penicillin. K562-A2 cells, expressing the HLA-A*02:01 molecule were cultured in the presence of 0.5 μg/ml of G-418, as previously described [41]. The T2 and K562-A2 cells were kindly provided by Dr. Pedro Romero (University of Lausanne) and by Dr Peter Ponsaerts (University of Antwerp, with the permission of Dr. Cedrik Britten from Johanes Gutenberg-University Mainz), respectively.

### Study populations

For the screening of the recognition of TcCA-2 epitopes by Chagas disease patients, 10 HLA-A*02:01 adult patients in the asymptomatic phase of the disease (IND) were enrolled. For cytotoxicity, functional activity determinations and phenotypic characterization another group of 19 HLA-A*02:01 adults Chagas disease patients and 9 healthy donors were analyzed. These patients were at the chronic stage of the disease and were considered to be at the asymptomatic phase (IND, n = 8) if there was no evidence of having cardiac or gastrointestinal disorders. Chronic Chagas cardiomyopathy patients (CCC, n = 11) were stratified into G1 to G3 stages following Kuschnir classification according to clinical criteria and radiological, electrocardiographic and echocardiography analyses. The number of patients was the one recommended by the Hospital Ethical Committee. The committee suggested that the amount of patients to be included in the study should be the minimal number that provides a statistically significant result. The patients were enrolled in both studies (functional and phenotypic characterization, IND n = 6 and CCC n = 7) when the available number of purified PBMC (limited by the cellular number isolated from each subject) was enough for both analyses. Another four CCC patients had to be enrolled to perform the phenotypic analysis in order to reach statistical significance.

These patients were Spain-residents coming from endemic areas, diagnosed for Chagas disease following WHO criteria using two conventional serological tests (Chagas ELISA, Ortho Clinical Diagnosis and Inmunofluor Chagas, Biocientífica, Argentina) at Hospital Virgen de la Arrixaca (Murcia-Spain), and that had never received treatment for the disease. HLA-A2-0201 patients selection was made at random among those that were positive after serological tests. These patients were clinically characterized in detail. Thirteen mL of blood samples from each patient were collected in EDTA. Peripheral blood mononuclear cells (PBMC) were purified as previously described in [21], stored in iFCS with 10% DMSO and cryopreserved in liquid nitrogen until use. HLA-A genotyping was carried out using the RELI^TM^ SSO HLA-A Typing kit (Invitrogen).

### Ethical considerations

The protocols were approved by the Ethical Committees at Hospital Clínic, Hospital Virgen de la Arrixaca and Consejo Superior de Investigaciones Científicas (Spain). A signed informed consent was obtained from all individuals prior to their inclusion in the study.

### HLA-A*02:01 binding assay

HLA-A*02:01 surface stabilization test was carried out as described in [42]. Briefly, TAP-deficient T2 cells were loaded with different concentrations of each peptide, in duplicate in serum-free RPMI medium, and incubated overnight at room temperature. Afterwards, the cells were stained with a PE-labeled HLA-A2-specific antibody (BB7.2 clone, BD biosciences) and analyzed by flow cytometry. The high-affinity HLA-A*02:01-binding peptide HB-ENV_334–342_ (WLSLLVPFV) was included as internal standard [43]. Affinity data were calculated as the percentage of maximum complex stabilization, as described in [44]. The following equation was used: % of maximal stabilization = 100 x [(mean fluorescence with peptide)–(background mean fluorescence)]/[(mean fluorescence with 25 μM HBENV_335-343_ peptide)–(background mean fluorescence)]. Background value was obtained with cells incubated in the same conditions without peptide [42].

### Granzyme B secretion in peripheral blood mononuclear cells from Chagas disease patients

The frequency of Granzyme B (GzB) producing cells was evaluated by ELISPOT assays using cryopreserved PBMC from Chagas disease patients and healthy donors as control. This protocol was carried out as described in [20]. Briefly, 96-well PVDF membrane-bottom plates (Millipore) were pre-wetted with 35% ethanol and washed five times with PBS. Subsequently, plates were coated with 100 μL of anti-GzB (5 μg/mL) monoclonal antibody (Mabtech) and incubated overnight at 4°C. Plates were washed with PBS and incubated with 200 μL/well of blocking solution (RPMI-1640-10% FCS) at room temperature for 30 minutes. Afterwards, 5 × 10^4^ PBMC/well were added and incubated with 1 μM of each peptide for 30 h at 37°C with 5% CO_2,_ in triplicates. As a positive control, the PBMC were stimulated with 50 ng/ml phorbol 12-myristate 13-acetate (PMA, Sigma) and 500 ng/ml ionomycin (Sigma), or 10 μg/mL of phytohaemaglutinin (PHA, Sigma). Supernatants were harvested and aliquoted at -80°C for subsequent cytokine secretion profile determination. After five washes, 100 μL/well of a 1 μg/mL solution of biotinylated GzB-specific antibody (Mabtech) was added in 0.5% iFCS in PBS, and the plates were incubated for 2 h at room temperature. Wells were extensively washed with PBS and incubated with 100 μL/well streptavidin-alkaline phosphatase (Sigma) at dilution of 1:6000 for 1 h at room temperature. After washing (5x) with PBS, 50 μL/well of substrate solution (NBT-BCIP substrate, Sigma) were added. The wells were incubated for 20–30 min at room temperature in the dark. The reaction was stopped by rinsing the plates with cold tap water. The spots were visualized in an AXIO PLAN 2 Imagine microscope and quantified using KS ELISPOT software. The results are expressed as the number of peptide-induced spots forming cell (SFC) x10^6^ PBMC after subtracting the spot number of peptide un-stimulated PBMCs (basal response). The results were considered positive when a minimum of 20 SFCx10^6^ PBMCs and of at least over twofold basal spot numbers were detected.

### Cytokine-secretion tests

Cytokine secretion (IL-4, IL-10, IL-6, IFN-γ, TNF-α) was determined in the supernatants of PBMC from Chagas disease patients and healthy donors, after *in vitro* stimulation with 1 μM of each peptide for 30 h at 37°C in RPMI supplemented with 10% iFBS as explained before. The secretion profile was determined by using a bead-based multiplex immunoassay system (Bio-Plex, Bio-Rad), following the manufacturer's instructions. The response was considered positive if an increase in the secretion of cytokines was at least three folds higher than the secretion by un-stimulated cells (basal secretion level) and at least, 4 pg/mL for IL-4, 2 pg/mL for IL-10, 15 pg/mL for IL-6 and 5 pg/mL for IFN-γ and TNF-α. The positive control consisted of 50 ng/mL Phorbol 12 myristate 13-acetate (PMA) and 500 ng/mL ionomycin (Sigma). Cytokine levels were quantified in each sample by using the Bio-Plex Manager software 4.1 (Bio-Rad).

In the HLA-A*02:01 restriction assay, peptide-pulsed K562-A2 cells were used as antigen-presenting cells (APC). Plates were seeded with 20,000 peptide-pulsed K562-A2 [41] or K562 cells as a negative control. After washing of non-bound peptide, APC were incubated with 60,000 effector cells per well and the IFN-γ secretion test was performed as mentioned above.

### CD8^+^ T cell peptide-specific phenotypic characterization

**Fig 1:**
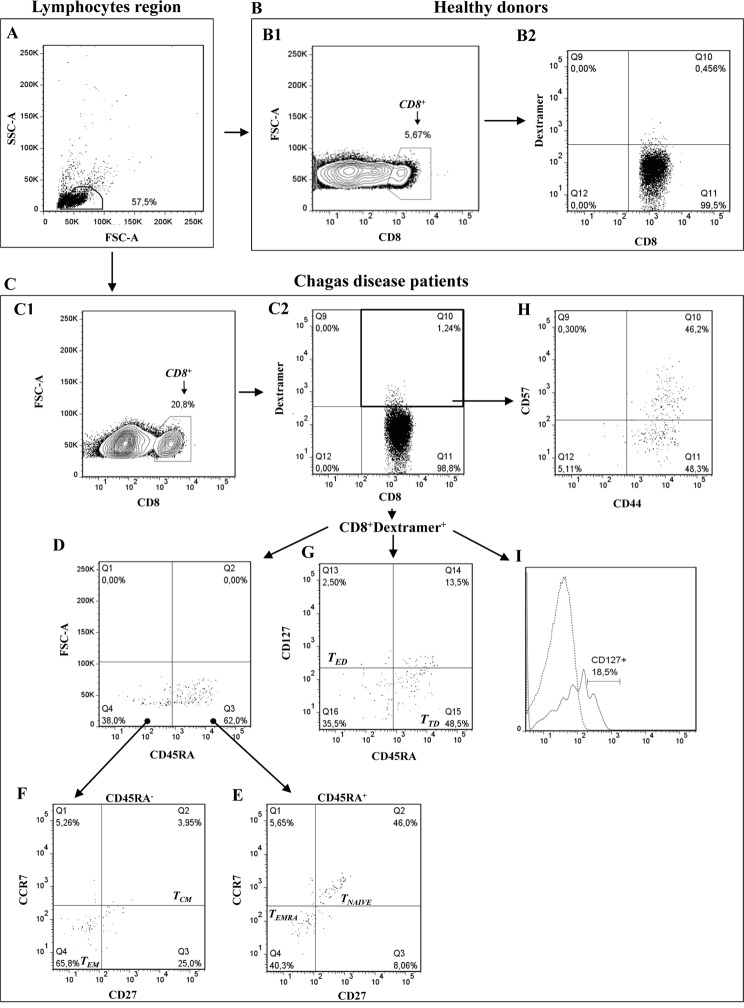
Phenotypic characterization of TcCA-2-specific CD8^+^ T cells. The dot plots show the lymphocyte gating based on forward-scatter (FSC) and side-scatter (SSC) properties (A), CD8^+^ T cell population (B1-C1) and the percentage of TcCA-2-specific CD8^+^ T cells (B2-C2) from a healthy control subject (B) and from chagasic disease patient (C) respectively. The phenotypic analyses of TcCA-2-specific CD8^+^ T cells, defined by CD45RA, CCR7, CD27, CD127, CD44 and CD57 expression (D-I) allowed to identify naive (CD45RA^+^CD27^+^CCR7^+^) and terminal effector memory (T_EMRA_, CD45RA^+^CD27^-^CCR7^-^) TcCA-2-specific CD8 T cells (E), central memory (T_CM_, CD45RA^-^CD27^+^CCR7^+^) and effector memory (T_EM_, CD45RA^-^CD27^-^CCR7^-^) TcCA-2-specific CD8^+^ T cells (F), early-stage differentiation (T_ED_, CD45RA^-^CD127^+^) and advanced-stage differentiation (T_TD_, CD8^+^CD45RA^+^CD127^-^) TcCA-2-specific CD8^+^ T cells (G), senescent memory (CD44^+^CD57^+^) and non-senescent memory (CD44^+^CD57^-^) TcCA-2-specific CD8 T cells (H) and CD27 expression in TcCA-2-specific CD8^+^T cells (I). Gates were established based on the control isotypes corresponding to each antibody. Non-stained cells and mononuclear cells incubated with antibodies but not with dextramers were used as reference to define the cut-off for each specific dextramer labeling.

TcCA-2-specific CD8^+^ T cells were characterized using a HLA-A*02:01 APC-labeled dextramers loaded with the TcCA-2_442-451_ and TcCA-2_607-615_ peptides, respectively (Immudex). 1X10^6^ PBMCs from 17 HLA-A*02:01^+^ Chagas disease patients and 6 healthy donors were incubated with 10μL of each dextramer in 40μL of 5% FCS in PBS for10 min at RT in the dark. Afterwards, these cells were incubated 20 min at 4°C with different cocktails of antibodies (BD Pharmigen): CD8-V500, CD8 PerCP-Cy5.5, CD45RA-APC-H7, CCR7-V450, CD27-FITC, CD127-PerCp-Cy5, CD57-FITC and CD44RA-APC. The labeled cells were washed twice with PBS-5% FCS and resuspended in 400 μL of PBS 1X. Data were acquired in a FacsAria III Cell Sorter and analyzed using Flowjo 7.6.5 software (Bio-Rad). At least 100.000 PBMC cells were acquired according to FCS/SSC parameters. The gating strategy is shown in Fig. 1.

### Statistical analysis

The statistical analysis was performed using Prism V5.0 (GraphPad Software, La Jolla, CA, USA). Nonparametric test were used to test for statistical significance. Comparison between asymptomatic and cardiac Chagas disease patients within the same cell subpopulation was evaluated by the Mann-Whitney U test (pairwise comparisons). In addition, Kruskal—Wallis test with Dunn correction was used to identify groups of cellular subpopulations or more than two groups of subjects (ANOVA for comparisons among ˃ 2 groups). Statistical significance was assigned at a value of p ≤ 0.05.

## Results

### Selection of HLA-A*02:01-binding peptides within *T*. *cruzi* TcCA-2 protein

In order to identify peptides containing potential HLA-A*02:01-binding sites, the deduced amino acid sequence of *T*. *cruzi* TcCA-2 was screened following the criteria described by Rammense et al [38] and using two computer algorithms. The SYFPEITHI and (IEDB) algorithms were employed to analyze binding affinity of the peptides to MHCI HLA-A*02:01 and the BIMAS to analyze the stability of the preformed complex. Eight peptides presented mid-to-high affinity score for HLA-A*02:01 (Table 1) and were consequently synthesized. All these peptides presented a low percentile rank score using the Immune Epitope Database and Analysis Resource (IEDB), which is associated to a high binding affinity (Table 1).

**Table 1:** Sequences of the TcCA-2-derived HLA-A*02:01 binding peptides.

Code	Position (aa)	Sequence ^a^	SYFPEITHI	BIMAS	IEDB	% maximal
peptide			score	score	score	stabilization
12828	TcCA-2 _273–281_	AAAGDKLSL *	23	0.29	18.00	12.41
12822	TcCA-2_442-451_	TVFDASRSTV	18	18.46	4.55	24.44
12823	TcCA-2_452-461_	FANAPGVAQV	22	10.22	6.50	46.23
12824	TcCA-2_489-498_	SILQNVHATL	26	10.86	3.80	36.02
12825	TcCA-2_531-540_	AIGGGKLPAL	28	6.75	6.90	20.89
12826	TcCA-2_550-559_	SAFGNHASTV	21	3.57	7.15	19.07
12827	TcCA-2_607-615_	ALRNLRVFL	24	8.92	6.80	29.54
12819	TcCA-2_657-666_	ALQVTNHRYL	22	23.49	7.00	21.49

The table shows the theoretical binding score to HLA-A*02:01 calculated by the computer algorithms SYFPEITHI and Immune Epitope Database and Analysis Resource (IEDB). The half time of complex disassociation (expressed in minutes) was calculated by the BIMAS algorithm. The maximal binding stabilization was calculated referred to HB-ENV_335-343_ peptide as a control.

^a^ Deduced amino acid from the sequence as found in GenBank, accession number: M92049.1. The sequence starts from the nucleotide 566 and ends on nucleotide 2461.

* The sequence encoding the peptide TcCA-2_273-281_ is contained in position 817–843 of the nucleotide sequence of the CL Brenner strain (accession number XM-813834).

The percentage of maximal binding stabilization of these eight TcCA-2 peptides was evaluated using TAP-deficient T2 cells and different concentrations of each peptide. The experimental results were referred with the fluorescence index values obtained with the HB-ENV_335–343_ peptide as it has been previously shown that this peptide has a high binding affinity to HLA-A*02:01 MHC I [43]. The maximal binding affinity is shown for each peptide in Table 1.

### Detection of CD8^+^ T lymphocytes specific for HLA-A*02:01^+^

In order to analyze whether the Chagas disease patients CD8^+^ T cells recognize the TcCA-2-derived peptides in the context of the HLA-A*02:01^+^ molecule, peptide-pulsed K562-A2 cells were used as APC in a multiplex immunoassay for detection of IFNγ secretion (Table 2). PBMC from HLA-A*02:01^+^ asymptomatic Chagas disease patients (IND) recognized four TcCA-2 epitopes (TcCA-2_657-666_, TcCA-2_442-451_, TcCA-2_607-615_ and TcCA-2_273-281_) while being presented on K562-A2 but not on K562 cells, which do not express Class I molecules. Two out of 9 patients responded to the TcCA-2_657-666_ peptide, 2 out of 10 to the TcCA-2_442-451_ peptide, 3 out of 9 to the TcCA-2_607-615_ peptide and 1 out of 10 to TcCA-2_273-281_.

**Table 2:** Recognition of TcCA-2 epitopes by HLA-A*02:01 Chagas disease patients.

Peptide	Chagas disease patient^a^	INF-γ secretion range (pg/mL)^b^
TcCA-2_273-281_	1/10	8.6
TcCA-2_442-451_	2/10	5.8–6.9
TcCA-2_452-461_	0/10	-
TcCA-2_489-498_	0/8	-
TcCA-2_531-540_	0/9	-
TcCA-2_550-559_	0/9	-
TcCA-2_607-615_	3/9	6.4–15.1
TcCA-2_657-666_	2/9	6.1–6.7

K562-A2 cells were pulsed with 1 mM of each peptide for 1 h, and co-cultured with PBMCs from asymptomatic HLA-A*02:01 Chagas disease patients. Following 30 h of growth, IFN-γ secretion was measured in the supernatants. IFN-γ secretion level in K652 cells, not expressing the class I molecule, was subtracted from the values obtained in the assays with the K562-A2 cells loaded with peptides.

Responses were considered positive when the supernatant IFN-γ concentration of the peptide-stimulated cells was at least two folds the concentration of the un-stimulated cells (non-peptide).

^a^ Patients with positive response, in relation with the total number of patients per assay.

^b^ Range of IFN-γ secretion by patients with a positive response to TcCA-2 peptides.

The cytotoxic activity of the CD8^+^ T cells specific for these four TcCA-2-derived epitopes was evaluated by secretion of GzB through ELISPOT assays. Thus, PBMC from 15 HLA-A*02:01 chagasic patients (8 IND and 7 CCC) and 9 healthy donors were incubated with the peptides under study. A positive GzB response was detected in 3 patients (2 IND and 1 CCC) out of 10 in response to incubation with the TcCA-2_657-666_ peptide, 5 patients (3 IND and 2 CCC) out of 14 in response to the TcCA-2_442-451_ peptide, 3 patients (3 CCC) out of 14 in response to the TcCA-2_607-615_ peptide and 2 patients (2 IND) out of 11 in response to the TcCA-2_273-281_ peptide (Table 3). Cytotoxic activities were not detected in any healthy donor.

**Table 3:** Cytokine-secretion and Granzyme B response to TcCA-2 epitopes by PBMCs from HLA-A*02:01 Chagas disease patients and healthy donors.

Clinical	Code	IL6	IFNγ	TNFα	GzB^c^	Clinical	Code	IL6	IFNγ	TNFα	GzB^c^
form^a^	patient	(pg/mL)^b^			(SFC/10^6^)	form^a^	patient	(pg/mL)^b^			(SFC/10^6^)
**TcCA-2**_**273-281**_						**TcCA-2**_**607-615**_					
HD	340	9	0	4	0	HD	340	6	0	0	0
HD	008	9	-1	0	-47	HD	008	22	3	0	-70
HD	664	0	-3	0	0	HD	664	0	-3	0	0
HD	001	1	6	-8	1	HD	001	-6	1	-14	0
HD	006	1	6	2	-7	HD	006	69	**13**	5	7
HD	005	-183	-19	-11	-47	HD	005	-77	-21	-2	-47
HD	004	-9	0	0	0	HD	004	-5	0	0	-7
HD	011	-11	0	-8	-20	HD	011	-12	0	-8	-7
IND	571	0	0	0	**67**	IND	535	-5	0	-1	-7
IND	535	-5	0	-1	-27	IND	265	**1127**	**81**	**83**	-100
IND	388	36	2	7	**33**	IND	283	**55**	**15**	**26**	-20
IND	365	**43**	1	-6	7	IND	388	32	1	-11	13
IND	510	1	0	-1	-20	IND	365	23	5	23	7
IND	917	18	7	11	13	IND	510	-1	0	2	13
CCC I	861	-4	2	-2	8	IND	917	-2	1	-3	-20
CCC I	884	0	0	0	7	CCC I	861	-7	0	-3	-8
CCC I	456	0	0	0	13	CCC I	884	0	0	0	0
CCC I	770	**22**	0	4	-7	CCC I	456	**18**	3	**12**	13
CCC III	509	-4	0	-5	0	CCC I	770	**49**	0	16	0
**TcCA-2**_**442-451**_						CCC II	142	-4	0	-15	**53**
HD	340	11	2	3	1	CCC III	797	9	-5	3	**27**
HD	008	31	1	15	-73	CCC III	509	-5	0	-4	**25**
HD	664	0	-3	0	1	**TcCA-2**_**657-666**_					
HD	001	18	4	-6	0	HD	340	22	1	8	1
HD	006	-2	2	2	-7	HD	008	3	-3	-21	-47
HD	005	16	5	-1	-40	HD	664	0	-3	0	0
HD	004	-15	0	-1	0	HD	017	0	0	0	0
HD	011	**54**	**8**	10	-20	HD	001	1	-2	-6	0
IND	535	-6	0	-1	-13	HD	006	-5	3	1	0
IND	265	**1641**	**204**	**160**	-13	HD	005	-58	0	-4	-40
IND	283	**80**	**12**	**35**	**47**	HD	004	-12	4	-1	-7
IND	388	21	1	-10	0	HD	011	-4	0	0	-7
IND	365	7	0	15	**27**	IND	571	0	1	0	**83**
IND	510	1	0	1	0	IND	535	-5	0	-2	**67**
IND	917	-13	-1	-4	**40**	IND	388	2	2	-5	7
CCC I	861	-4	0	-3	0	IND	365	10	2	14	0
CCC I	884	0	0	0	7	IND	510	-3	0	1	-7
CCC I	456	**46**	4	**6**	-7	IND	917	-17	-1	-17	-10
CCC I	770	3	-2	-11	13	CCC I	861	-4	0	-2	**50**
CCC II	142	-3	2	-20	**80**	CCC I	884	0	0	0	0
CCC III	797	**31**	3	**7**	7	CCC I	770	1	0	-6	-7
CCC III	509	-4	0	-5	**50**	CCC III	509	-4	0	-5	0

^a^ HLA-A*02:01 Chagas disease patients in asymptomatic phase (IND) and cardiac forms (CCC). HD: HLA-A*02:01 healthy donors. The cytokine secretion and the number of GzB-secreting cells were calculated by subtracting background levels or the number of spots in control wells (non-peptide) from the levels measured or the number of spots obtained in the stimulated cultures. Responses were considered positive (in bold) when:

^b^ The cytokine concentration is at least three folds the non-peptide control value and ≥15 pg/mL for IL-6 and ≥ 5 pg/mL for IFN-γ and TNF-α.

^c^ The number of SFC per million was ≥20 and at least twice the value found in the background control wells.

Functional analysis of the epitope-specific CD8^+^ T cells was carried out by the determination of the secretion profile of IFN-γ, TNF-α, IL-4, IL10 and IL-6 cytokines. The results, presented in Table 3, show that CD8^+^ T cells from 2 out of 14 patients recognized peptides TcCA-2_442-451_ by secreting IFN-γ, TNF-α and IL-6 and 2 out of 14 by secreting TNF-α and IL-6. The TcCA-2_607-615_ peptide was recognized by 2 out of the 14 patients cells, by secreting IFN-γ, TNF-α and IL-6, 1 out 14 by secreting TNF-α and IL-6 and 1 out 14 by secreting IL6. CD8^+^ T cells from 2 out of 11 patients responded to stimulation with peptide TcCA-2_273-281_ by secreting IL-6. Any production of cytokines was detected after stimulation with the TcCA-2_657-666_ peptide. There was no secretion of IL-4 and IL-10 in PBMC after stimulation with any one of the four TcCA-2 peptides (data not shown). IFN-γ, TNF-α and IL6 cytokines secretion levels were higher in patients at the asymptomatic phase of the disease than in Chagas patients with associated cardiomyopathy.

### Phenotypic characterization of T CD8^+^ lymphocytes specific for TcCA-2 derived peptides

**Fig 2:**
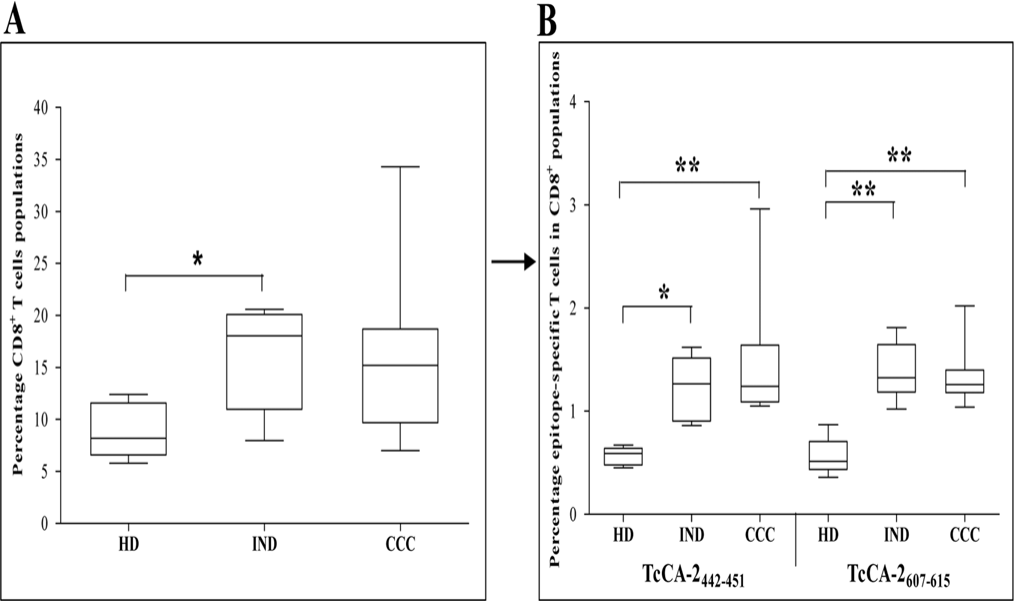
TcCA-2_442-451_ and TcCA-2_607-615_ CD8^+^ specific T cells. (A), percentage of CD8^+^ T cells in the PBMC region (see Fig. 1C1). (B), peptide-specific cells of total CD8^+^ T cells. Cell subpopulations were determined by flow cytometry in 6 healthy donors (HD), 6 patients in the asymptomatic phase (IND) and 11 patients in the cardiac phase (CCC). Median values are represented by horizontal lines. Error bars represent standard deviation intervals. Statistical analyses were carried out using Kruskal—Wallis test with Dunn correction (*). Statistically significant differences are indicated ^(^*^)^ p≤0.05, ^(^**^)^ p≤0.01, ^(^***^)^ p≤ 0.001.

After having shown the lymphocyte activation capacity of TcCA-2-derived epitopes (TcCA-2_657-666_, TcCA-2_442-451_, TcCA-2_607-615_, and TcCA-2_273-281_) we decided to phenotypically characterize, the specific CD8^+^ T lymphocytes that recognized the two peptides that exhibited the greatest recognition by specific CD8^+^ T cells (TcCA-2_442-451_ and TcCA-2_607-615_) (scheme in Material and Methods Section, Fig. 1). With that aim, mononuclear cells from Chagas patients in different phase of the disease (IND, n = 6 and CCC, n = 11) as well as from healthy donors (HD, n = 6) were incubated with HLA-A*02:01 APC-labeled dextramers loaded with the TcCA-2_442-451_ and-TcCA-2_607-615_ peptides. As shown in Fig. 2A, the percentage of total CD8^+^ T lymphocyte in the PBMC gate was significantly higher in asymptomatic Chagas disease patients (IND) than in healthy donors (p≤0.05). In addition, we observed that IND and CCC Chagas disease patients had a higher percentage of TcCA-2_442-451_ and TcCA-2_607-615_ specific CD8^+^ T cells than healthy individuals (p≤0.05 and p≤0.01) (Fig. 2B).

**Fig 3:**
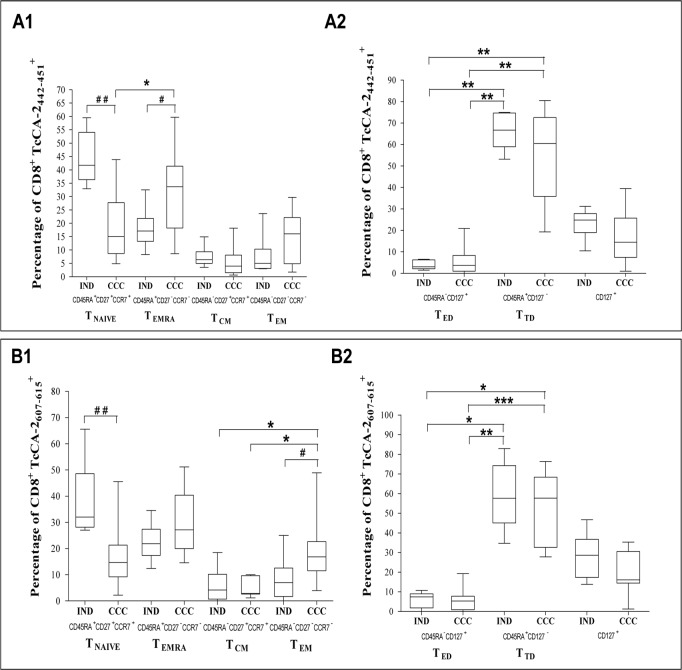
Immunophenotyping of memory and differentiation status of the specific CD8^+^ T cells in patients with Chagas disease. (A) TcCA-2_442-451_ peptide. (B) TcCA-2_607-615_ peptide. PBMCs from 6 asymptomatic form patients (IND) and 11 patients with the cardiac form (CCC) were stained for CD45RA, CD27 and CCR7 and analyzed by flow cytometry. According to the combination of antibodies used the CD8 peptide-specific cells were divided in: T_NAIVE_ (CD8^+^CD45RA^+^CD27^+^CCR7^+^), T_EMRA_ (CD8^+^CD45RA^+^CD27^-^CCR7^-^), T_CM_ (CD8^+^CD45RA^-^CD27^+^CCR7^+^), T_EM_ (CD8^+^CD45RA^-^CD27^-^CCR7^-^), T_ED_ (CD8^+^CD45RA^-^CD127^+^), T_TD_ (CD8^+^CD45RA^+^CD127^-^). Median values are represented by horizontal lines. Error bars represent standard deviation intervals. Statistical analyses were carried out using Mann-Whitney U test (#) and Kruskal—Wallis test with Dunn correction (*). Statistically significant differences are indicated ^(#)^ or ^(^*^)^ p≤0.05, ^(# #)^ or ^(^**^)^ p≤0.01, ^(# # #)^ or ^(^***^)^ p≤ 0.001.

Labeling, using antibodies against CD45RA, CD27 and CCR7 molecules, allowed us to phenotypically characterize TcCA-2_442-451_ and TcCA-2_607-615_-specific CD8^+^ T cells from patients at different phases of the disease (IND and CCC). Thus, as shown in Fig. 3A_1_ we observed that the TcCA-2_442-451_-specific CD8^+^ T cells from IND patients have a higher percentage of cells expressing the T_NAIVE_ phenotype (CD45RA^+^CD27^+^CCR7^+^) than the cells from CCC patients (p≤0.01). However, the percentage of terminal effector memory CD8^+^ T cells (T_EMRA_ cells, CD45RA^+^CD27^-^CCR7^-^) was significantly lower in IND patients than in CCC (p≤0.05) (Fig. 3A_1_). Moreover, the TcCA-2_442-451_-specific CD8^+^ T cells from CCC patients have a higher percentage of T_EMRA_ cells than that having a phenotype T_NAIVE_ (p≤0.05). Regarding the TcCA-2_607-615_-specific CD8^+^ T cells, the percentage of cells expressing CD45RA^+^CD27^+^CCR7^+^ (T_NAIVE_) was also significantly higher in IND *versus* CCC patients (p≤0.01) (Fig. 3B_1_). The percentage of T_EM_ is higher in CCC patients than that in IND patients (p≤0.05). Moreover, the percentage of T_EM_ *versus* of T_CM_ cells is also higher in CCC patients than that in IND patients (p≤0.05). In fact, the TcCA-2_607-615_-specific CD8^+^ T cells from CCC patients had mainly an effector phenotype showing a predominant profile of T_EM_ cells *versus* of T_CM_ cells (p≤0.05) (Fig. 3B_1_). By combining the CD45RA and CD127 surface markers it was observed (Fig. 3A_2_ and 3B_2_) that both IND and CCC patients have a higher percentage of TcCA-2_442-451_ and TcCA-2_607-615_—specific CD8^+^ T cells at an advanced-stage differentiation (T_TD_, CD45RA^+^CD127^-^) phenotype than at an early-stage differentiation (T_ED,_ CD45RA^-^CD127^+^) phenotype (p≤0.01).

**Fig 4:**
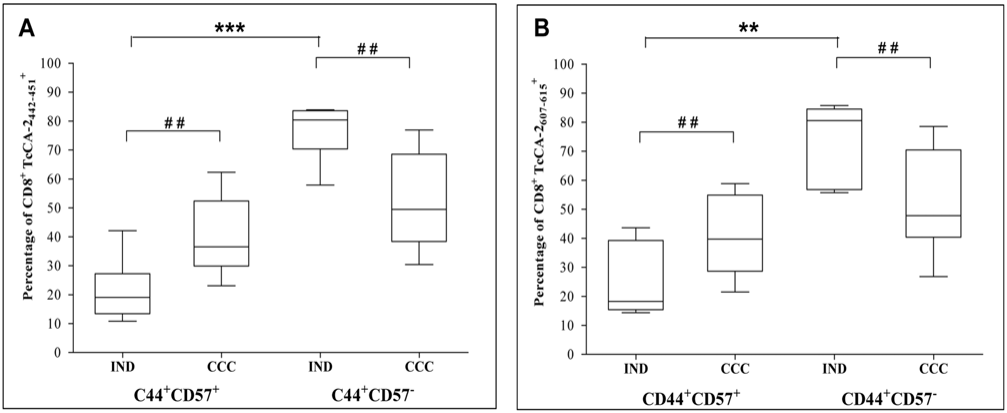
Expression of the senescence marker CD57 in antigen-experienced specific CD8^+^ T cells. (A) TcCA-2_442-451_-specific T cells. (B) TcCA-2_607-61_-specific T cells. Cells from 6 patients in asymptomatic phase (IND) and 11 patients with the cardiac form (CCC) were analyzed. Median values are represented by horizontal lines. Error bars represent standard deviation intervals. Statistical analyses were carried out using Mann-Whitney U test (#) and Kruskal—Wallis test with Dunn correction (*). Statistically significant differences are indicated ^(#)^ or ^(^*^)^ p≤0.05, ^(# #)^ or ^(^**^)^ p≤0.01, ^(# # #)^ or ^(^***^)^ p≤ 0.001.

Since the CD57 expression is associated with replicative senescence we evaluated the CD57 expression in CD44^+^ cells (marker of antigen-experience T cells) of both TcCA-2_442-451_ and TcCA-2_607-615_-specific T CD8^+^ cells. The results shown in Fig. 4A and 4B indicate that in IND patients the predominant phenotype is of non-senescent memory cells (CD44^+^CD57^-^, p≤0.001) and that the percentage of circulating senescent memory cells (CD44^+^CD57^+^) specific for both epitopes is significantly higher in CCC patients than that in IND patients (p≤0.01).

## Discussion

CD8^+^ T cells are essential for controlling *T*. *cruzi* infection [45]. Nevertheless, parasite-derived antigens capable of inducing CD8^+^ T cells activation have been characterized mainly in mouse models [46–48]. By performing an *in silico* assessment with two independent bioinformatic algorithms we have identified eight epitopes that theoretically bind to HLA-A*02:01 class I MHC molecules in the *T*. *cruzi* protein TcCA-2. The identification of HLA-A*02:01-binding epitopes was carried out using peptide—pulsed K562-A2 cells as APCs in an IFN-γ secretion test. The K562-A2 cell line is an inexhaustible source of APCs [41] and efficiently presents HLA-A*02:01 binding peptides to specific CD8^+^ T cells showing a low level of background reactivity [49]. The data obtained showed that 4 out of the 8 theoretically HLA-A*02:01-binding epitopes were recognized, processed and presented in the context of a natural *T*. *cruzi i*nfection. The use of a cellular line as APC instead of the “natural” presenting cells that are present in the PBMC may influence the percentage of patients that recognized the different peptides. Taking into account the secretion level of GzB and IL-6, IFN-γ and TNFα cytokines a two fold increase of the number of responsive patients was observed when the own patient´ APC were employed. Interestingly, CD8^+^ T cells specific for these four epitopes are functionally active in both the asymptomatic (IND) and the symptomatic (CCC) Chagas disease chronic patients showing a differential phenotype depending on the severity of the disease. The identification of new class I-restricted epitopes are helpful for designing biomarkers of sickness pathology as well as the development of immunotherapies against *T*. *cruzi* infection. The fact that only a fraction of patients responded to particular peptides may be a consequence of the existence of high variability regarding the antigen processing and presentation by the Chagas disease patients. Consequently, the use of several epitopes belonging to others *T*. *cruzi* antigens will be required to perform an accurate disease follow-up. In fact, an increase in the number of responsive patients and in particular an increase in the multifunctional response capacity (both cytokines secretion and cytotoxic activity) of the epitope-specific CD8^+^ T cells was detected (data not shown), when the response against CD8^+^ T epitopes belonging to other *T*. *cruzi* antigens [20,21] was considered.

The HLA-A*02:01-binding *in vitro* assays using the TAP-deficient T2 cells shows that the correlation between the theoretical and the *in vitro* binding data is low. Similarly, low-medium correlation coefficients have been described between experimental and theoretical HLA-A*02:01-binding data for other *T*. *cruzi* antigens [21,50]. The identification of class I epitopes has been limited to the HLA-A*02:01 molecule due to the fact that it is the most prevalent human Class I allele [51].

The TcCA-2–specific CD8^+^ T cells from chronic patients at the asymptomatic and cardiac phases of the disease have cytotoxic activity. Remarkably, CD8^+^ T cells specific for the TcCA-2_442-451_ and TcCA-2_607-615_ epitopes also secreted the pro-inflammatory cytokines IFN-γ and TNFα. The secretion of these cytokines has been correlated to an improved protective CD8^+^-mediated immunity against *T*. *cruzi* infection [14,52]. In addition, the TcCA-2_442-451_, TcCA-2_607-615_ and TcCA-2_273-281_–specific CD8^+^ T cells from chronic patients secreted IL6 cytokine. The IL-6 is a pleiotropic cytokine related to inhibition of expansion and functionality of Treg lymphocytes [53]. Secretion of anti-inflammatory cytokines (IL-4 and IL-10) was not detected in any of the samples isolated from the patients evaluated in the present study after TcCA-2—epitopes stimulation.

The experiment carried out with labeled dextramer loaded with the TcCA-2_442-451_ and TcCA-2_607-615_ peptides showed that Chagas patients in both IND and CCC phase presented a similar percentage of epitope-specific CD8^+^ T cells. A combined use of these dextramers with different surface markers shows the existence of a differential phenotypical profile in cells from IND *versus* CCC Chagas patients. Thus, IND Chagas patients present a significantly higher amount of TcCA-2_442-451_ and TcCA-2_607-615_ epitope-specific CD8^+^ T cells with a T_NAIVE_ phenotype than cardiac phase patients. However, in cardiac chronic patients there is a predominant effector memory (T_EM_) phenotype or a terminally differentiated effector CD8^+^ T cells (T_EMRA_) phenotype. The effector phenotype observed in epitope-specific CD8^+^ T cells isolated from CCC patients does not correspond to an effector response, characterized by cytokine production. In fact, our results show that the TcCA-2 epitope-specific CD8^+^ T cells isolated from patients undergoing asymptomatic phases secreted higher IFN-γ and TNF-α than the TcCA-2 epitope-specific CD8^+^ T cells isolated from symptomatic patients. A higher clinical severity has been described in patients with a low count of *T*. *cruzi* antigen specific IFN*-γ* producing CD8^+^ T cells [54,55]. Likewise, similar results have been obtained in experimental *T*. *cruzi* chronic infection mouse models, in which most of the CD8^+^ T cells infiltrated in the cardiac tissue express surface markers of an effector memory phenotype although its effector activity in response to antigenic activation remains poor [56]. This fact can be a consequence of a functional alteration of these lymphocytes associated with the symptomatic status of the disease. The alteration in the antigen-specific CD8^+^ T cells response may lead to a progression of the disease related to the persistence in the host and the parasite load. It is known that a persistent infection leads to a progressive functional alteration of the CD8^+^ T cells immune response characterized by a lack of proliferation capacity, low expression of IL-7 receptor (CD127), as well as a reduced TNF-α and IFN-γ production [57,58].

Our results show that in IND and CCC patients the percentage of fully differentiated epitope-specific CD8^+^ T cells having a CD127^-^/CD45RA^+^ phenotype is higher than that having an early differentiation CD127^+^/CD45RA^-^ phenotype. Furthermore, the percentage of epitope-specific CD8^+^ T cells with CD127^+^ phenotype is lower in CCC patients than in IND patients. CD127 is a marker that identifies memory CD8^+^ T cells precursors capable of generating long lived antigen-independent memory CD8^+^ T cells [30]. Moreover, it has been shown that prolonged antigen exposure during chronic parasitic, viral and bacterial infections results in failure of memory CD8^+^ T cells able to acquire the properties of antigen independent memory T cells [31,57]. The analysis of the CD57^+^ and CD44^+^ surface markers associated with clonal senescence [59] and antigen experience [60], respectively, shows the existence of a significantly higher percentage of senescent CD8^+^ T cells in CCC patient than in IND patients specific for both TcCA-2_442-451_ and TcCA-2_607-615_ epitopes. In summary, our data suggest that there is a gradual clonal exhaustion associated with increased disease severity, which might be the result of continuous antigenic stimulation by persistent parasites. In the chronic phase of Chagas heart disease it has been suggested that parasite persistence and immunological mechanisms are inextricably related to the myocardial aggression [9,61]. However, it has also been described that there is heart tissue damage in the absence of detectable live parasites [62]. We observed that 57% of the Chagas disease patients included in the analysis were PCR positive (data not shown). Currently, we are in the process of analyzing the dynamic of the phenotypic profile and functionality of TcCA-2 epitope-specific CD8^+^ cells after treatments with benznidazole in a large number of infected subjects at different stages of the disease.
